# Unusual Painful Muscle Spasm in a Dialysis-Dependent Patient with Progressive Cervical Kyphosis

**DOI:** 10.1155/2019/7062568

**Published:** 2019-02-20

**Authors:** Daisuke Tsunoda, Yoichi Iizuka, Tokue Mieda, Haku Iizuka, Hirotaka Chikuda

**Affiliations:** ^1^Department of Orthopaedic Surgery, Gunma University Graduate School of Medicine, 3-39-22 Showa, Maebashi, Gunma 371-8511, Japan; ^2^Department of Orthopaedic Surgery, Isesaki Municipal Hospital, 12-1 Tsunatori Honmachi, Isesaki, Gunma 372-0817, Japan

## Abstract

We herein report a case of cervical myelopathy due to destructive spondyloarthropathy (DSA) with an unusual painful muscle spasm. A 51-year-old man presented to our hospital due to numbness in the hands and progressive gait disturbance. The patient underwent laminoplasty for cervical myelopathy. One and a half years after surgery, the patient developed progressive kyphosis of the cervical spine and increasing spastisity. He also started to suffer from intractable pain associated with a muscle spasm. A blood analysis revealed marked CK elevation. The patient underwent 2-stage circumferential decompression and fusion. After the revision procedure, his symptoms improved, and his serum CK level normalized. Physicians should be alert to the possibility of pain associated with an excessive muscle spasm in cervical myelopathy patients.

## 1. Introduction

Patients with cervical myelopathy may present with a variety of manifestations, including increased tonus of the skeletal muscles. However, there are no previous reports of cervical compressive myelopathy in which the main manifestation is a painful muscle spasm.

We herein report a case of cervical myelopathy due to destructive spondyloarthropathy (DSA) with a severe painful muscle spasm.

## 2. Case Report

**Figure 1:**
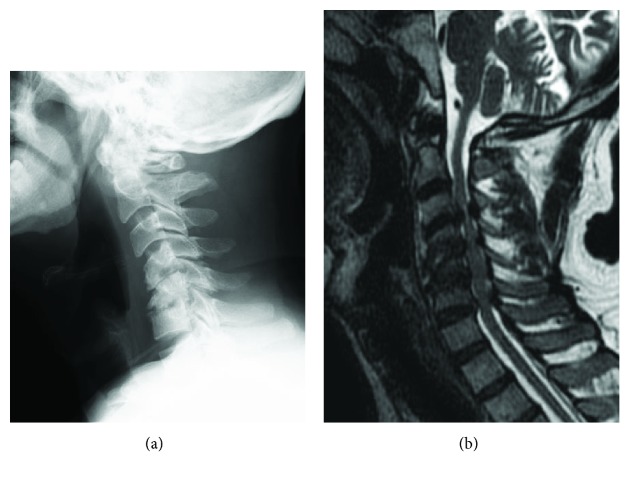
(a) X-ray of the cervical spine obtained just before the initial surgery showed destructive changes of the cervical spine, with a C2-7 lordotic angle of 0°. (b) Sagittal T2-weighted MRI of the cervical spine performed just before the initial surgery showed multisegmental stenosis at the C3/4, C4/5, C5/6, and C6/7 disc levels.

A 51-year-old man presented to our hospital due to symptoms of myelopathy. He had been undergoing hemodialysis due to chronic kidney failure associated with nephrotic syndrome for over 10 years. He complained of numbness in the extremities and clumsy hands, and he was unable to walk without assistance. Spastic gait disturbance associated with increased muscle tonus was observed, and his serum CK level remained slightly high (315 U/l). Cervical laminoplasty was performed for cervical myelopathy related to cervical DSA (Figure 1). The postoperative course was uneventful. His numbness and clumsy hands improved, and he became ambulatory.

Two months after the initial surgery, however, his condition started to deteriorate. He developed unusual intractable pain throughout his whole body, and cramp-like muscle pain was observed paroxysmally and frequently with severe spasticity. Regarding the pain intensity, the numerical rating scale (NRS) score (wherein 0 = no pain and 10 = the worst pain), painDETECT score [1], and neuropathic pain symptom inventory (NPSI) [2] were 10, 28, and 79, respectively.

**Figure 2:**
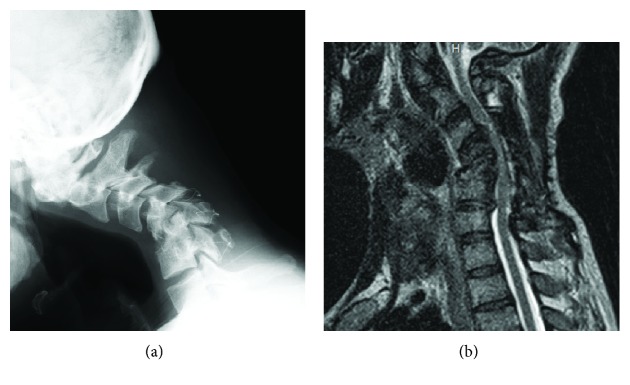
(a) X-ray of the cervical spine obtained 1 year after the initial surgery revealed progression of the destructive changes, with a C2-7 lordotic angle of -53°. (b) Sagittal T2-weighted MRI of the cervical spine performed one year after the initial surgery showed that the spinal cord was compressed by multisegmental stenosis with kyphotic deformity.

**Figure 3:**
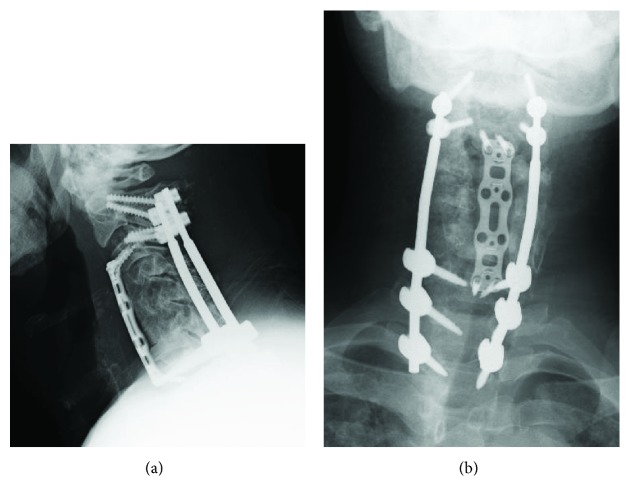
X-ray of the cervical spine obtained just after the two-stage additional surgery showed restoration of the cervical alignment by posterior and anterior spinal fusion surgery.

While his pain was partially relieved by the administration of ketamine, his symptoms were disabling and not sufficiently managed by conservative treatment. Plain radiographs showed the progression of destructive changes at the C4/5 and C5/6 levels. A marked progression of kyphosis of the subaxial spine was noted with a C2-7 angle of -53° (Figure 2). A laboratory examination revealed that his serum level of CK was extremely high (999 U/l). With a marked elevation of CK, we first consulted neurologists and nephrologists regarding the possible underlying pathology. The differential diagnosis included myopathy, an electrolyte imbalance, and an adverse drug reaction; however, the cause of the patient's condition remained unclear. Therefore, we performed additional surgery to resolve the deteriorated destructive changes in the cervical spine, which we assumed to be potentially responsible for his symptoms. In the first stage of surgery, cervicothoracic posterior spinal fusion was performed from C2 to T2 using pedicle screws at the C2, C3, C7, T1, and T2 vertebral levels. In the second stage of surgery (10 days after the first stage), anterior spinal fusion was performed from C3 to C7 with an autologous iliac bone graft and a titanium plate (Figure 3).

**Figure 4:**
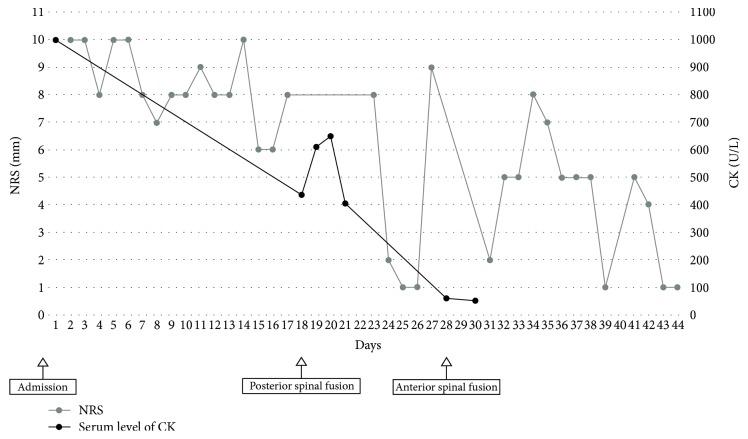
The time courses of the numerical rating scale score and serum value of creatinine kinase before and after two-stage surgery.

The patient's intractable pain disappeared within 2 weeks after surgery. On comparing the pain intensity between before and after the two-stage surgery, the NRS score improved from 10 to 1, the painDETECT score changed from 28 to 10, and the NPSI score changed from 79 to 10. The serum level of CK was also normalized after the two-stage surgery (Figure 4). At 10 months after surgery, he was able to walk without any support. The patient and his family were informed that data from the case would be submitted for publication and gave their informed consent.

## 3. Discussion

Our patient had debilitating pain associated with a severe muscle spasm, an uncommon manifestation of cervical compressive myelopathy, which was triggered by subtle stimulation. Marked serum CK elevation (approximately 1,000 U/l) suggested muscle tissue damage caused by excessive muscle spasm. Of note, the patient's serum CK level was normalized once the painful muscle spasms were resolved after revision surgery. To our knowledge, there have been no reports regarding the association between cervical compressive myelopathy and the increase in serum CK due to spasticity.

Colachis and Rea reported a case of T4 paraplegia with recurrent episodes of severe spasticity and CK elevation associated with intrathecal baclofen (ITB) withdrawal. The authors hypothesized that the elevated CK level, as a clear indication of severe hyperexcitability and contractibility of muscle fibers in muscle breakdown, was associated with a severe increase in spasticity due to ITB withdrawal [3]. Similarly, in the present case, we assumed that the excessive muscle contraction associated with spasticity resulted in the dissolution of muscle and marked CK elevation.

It is widely accepted that patients with cervical cord compression often present with neuropathic pain [4]. The changes of painDETECT and NPSI scores indeed suggest that neuropathic pain played a role in the present case as well. However, severe damage to muscle tissue—as evidenced by the patient's CK elevation—indicated a distinct pathomechanism besides neuropathic pain in this case. Interestingly, a similar symptom known as painful tonic spasm has been reported in patients with various neurological disorders, including multiple sclerosis [5] and neuromyelitis optica [6]. Further studies and the accumulation of more case reports are warranted to clarify the pathomechanism of a painful muscle spasm in cervical compressive myelopathy patients. Physicians should be alert to the possibility of pain caused by an excessive muscle spasm in cervical myelopathy patients, in particular for those with severe spasticity.
